# A Cross‐Sectional Examination of Movement Behaviours and Guideline Adherence Rates Among Preschool‐Aged Children With Disabilities

**DOI:** 10.1111/cch.70088

**Published:** 2025-04-28

**Authors:** Maeghan E. James, Kathleen A. Martin Ginis, Rebecca L. Bassett‐Gunter, Krista L. Best, Kelly P. Arbour‐Nicitopoulos

**Affiliations:** ^1^ Healthy Active Living and Obesity Research Group, Children's Hospital of Eastern Ontario Research Institute Ottawa Canada; ^2^ Department of Medicine University of British Columbia Vancouver British Columbia Canada; ^3^ School of Health and Exercise Sciences University of British Columbia Kelowna British Columbia Canada; ^4^ School of Kinesiology and Health Science LaMarsh Centre for Child and Youth Research, York University Toronto Ontario Canada; ^5^ Département de réadaptation Université Laval Québec Canada; ^6^ Faculty of Kinesiology and Physical Education University of Toronto Toronto Ontario Canada

**Keywords:** Childhood Disability, Health, Physical Activity, Preschool Children, Sleep

## Abstract

**Background:**

Meeting the 24‐h movement behaviour guidelines (hereafter ‘guidelines’) supports healthy growth and development. It is recommended that preschool‐aged children engage daily in 180 min of physical activity (PA), 60 min of which are moderate‐to‐vigorous PA (MVPA), along with less than 1 h of screen time and 10–13 h of sleep for optimal health. However, research on movement behaviours among children with disabilities in the early years is limited. This study aimed to describe movement behaviours and guideline adherence rates among preschool‐aged children with disabilities.

**Methods:**

A cross‐sectional subanalysis was conducted of caregivers of young children (aged 4 years) with a disability (*N* = 31; 65% boys, 55% with a developmental disability) who were part of a Canadian study on movement behaviours in children and youth with disabilities. Caregiver‐reported PA, screen time and sleep behaviours were collected using an online survey. Descriptive statistics were used to describe children's movement behaviours and proportion meeting the guidelines.

**Results:**

On average, children engaged in 108 min (SD = 89.9) of total PA and 40 min (SD = 47.0) of MVPA per day. Children engaged in an average of 3.5 h (SD = 2.4) of screen time per day and slept an average of 10.6 h (SD = 1.0) per night. Overall, 23.1%, 6.9% and 79.3% of children met the guidelines for PA, screen time and sleep, respectively. One child (4.3%) met all three guidelines.

**Conclusions:**

Few children with disabilities met the preschool‐aged PA and screen time guidelines, though most met the sleep guideline. These findings highlight the need to further examine adherence of movement behaviour guidelines among a more representative sample of young children with disabilities and factors influencing adherence.

**Summary**

- This study highlights a significant gap in meeting physical activity recommendations among preschool‐aged children with disabilities.
- Results demonstrated that screen time among preschool‐aged children with disabilities far exceeds recommended limits, highlighting a need for strategies to reduce sedentary behaviour in this population.
- Sleep was the most successfully adhered‐to guideline, indicating that children with disabilities generally meet the recommended sleep duration.
- These findings emphasize the need for increased surveillance research on movement behaviours among preschool‐aged children with disabilities in the early years.

## 1 Introduction

Engaging in more physical activity (PA), less sedentary behaviour (including screen time) and uninterrupted sleep can support holistic health and development in young children (Kuzik et al. 2017). Establishing healthy patterns of PA, sedentary behaviour and sleep in the early years is especially important for setting the foundation for healthy behaviours later in life (Telama and Hirvensalo 2015). Recognizing this, movement behaviour guidelines have been established by individual countries and the World Health Organization for children in the early years. In Canada, these daily guidelines recommend that preschool‐aged (i.e., 3–4 years) children engage in at least 180 min of PA at any intensity throughout the day, with at least 60 min spent in energetic play (i.e., moderate‐to‐vigorous PA; MVPA); engage in no more than 60 min of screen time; and have at least 10–13 h of good quality sleep (Tremblay et al. 2017).

Globally, most young children are meeting the age‐specific guidelines for PA (62%–93%) and sleep (63%–89%), and fewer are meeting the age‐specific sedentary behaviour guideline (2%–38%) or all three movement behaviour guidelines (7%–19%) (Chaput et al. 2017; Cliff et al. 2017; Nyström et al. 2020; Tanaka et al. 2023). Within these studies, little focus has been directed towards reporting on movement behaviours in young children with disabilities, thus limiting understanding of movement behaviour within this child population. Children with disabilities are defined as those who have long‐term physical, mental, intellectual and/or sensory impairments that prevent full and equal participation in society (United Nations 2008). In 2021, it was estimated that 29 million children globally between the ages of 0 and 4 years old had moderate‐to‐severe disabilities (United Nations Children's Fund 2021).

Several disparities have been reported for children with disabilities in the early years, including barriers to participation in community and childcare environments (Benjamin et al. 2016; United Nations Children's Fund 2021), highlighting a need for research exploring movement behaviours specifically among children in this population. This short communication addresses this gap by presenting the findings from the first study in Canada and, to our knowledge, internationally, which examines the three movement behaviours among children with disabilities in the early years.

## 2 Methods

A subanalysis of the larger National Physical Activity Measurement (NPAM) study was conducted to examine guideline adherence in a sample of 31 young children (aged 4 years) with disabilities. NPAM was a cross‐sectional online study conducted between January 2018 and May 2023 (with a temporary pause on data collection from March 2020 to July 2022 due to the COVID‐19 pandemic) to examine the movement behaviours of children and youth with disabilities aged 3–17 years old in Canada (Arbour‐Nicitopoulos et al. 2023). As part of the NPAM study, caregivers completed an online survey that took approximately 25 min to complete. Caregiver survey data pertaining to family demographics (e.g., age and disability type) and child movement behaviours (overall PA, MVPA, screen time and sleep) were used in the subanalysis.

PA, screen time and sleep were measured using items from the International Physical Activity Questionnaire for Children and Adolescents (Hagströmer et al. 2005), Health Behaviour in School‐aged Children (HBSC) survey (Roberts et al. 2009) and the International Study on Childhood Obesity, Lifestyle, and the Environment (ISCOLE) Diet and Lifestyle Questionnaire (Katzmarzyk et al. 2019), respectively. Details on the items used and how movement behaviour durations were calculated can be found in Appendix A. Data analysis was conducted using SPSS version 24.0. Descriptive statistics (*n*, %) were used to summarize sample demographic and child movement behaviour characteristics, and guideline adherence.

## 3 Results

**FIGURE 1:**
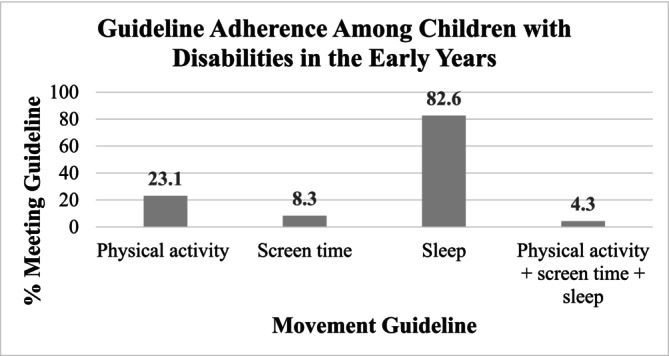
Children with disabilities' adherence rates to individual (i.e., 180 min of total PA and 60 min of MVPA; less than 2 h of screen time and 10–13 h of sleep) and combined movement behaviour guidelines in the early years.

Thirty‐one caregivers (87% mothers, mean age = 37 years, SD = 4 years) of preschool‐aged children with disabilities (*M*_age_ = 4 years, SD = 0) completed the survey. Table 1 presents a complete demographic description of these caregivers and their children. Table 2 presents the mean duration children were reported to spend in daily PA (including MVPA), screen time and sleep. On average, caregivers reported their child to be engaging in 108 min (SD = 90) of total PA per day, 40 (SD = 47) of which were spent in MVPA. Children were reported to engage in an average of 225 min (SD = 170) of daily screen time per day and 10.6 h (SD = 1 h) of sleep per night. Adherence rates for the three movement behaviour age‐specific guidelines are presented in Figure 1. Most children (83%) met the sleep guideline, with fewer meeting the guideline for PA (23%) or sedentary behaviour (8%). One child (4%) in the sample met the guideline for all three movement behaviours. For the PA guideline, 31% of children engaged in MVPA at least 60 min each day and 24% engaged in PA at any intensity for at least 180 min each day.

**TABLE 1:** Demographic characteristics of caregivers and children (*N* = 31).

Caregiver demographic profile	*n* (%)
Relationship to child, mother	27 (87.1)
Age (mean, SD)	37.2 (4.4)
Province	
British Columbia	14 (45.2)
Alberta	1 (3.2)
Manitoba	1 (3.2)
Ontario	9 (29.0)
Quebec	3 (9.7)
New Brunswick	1 (3.2)
Nova Scotia	1 (3.2)
Yukon Territory	1 (3.2)
Employment status	
Full‐time	11 (35.5)
Part‐time	5 (16.1)
Self‐employed	2 (6.5)
Unemployed, currently looking for work	1 (3.2)
Unemployed, not currently looking for work	8 (25.8)
Other (e.g., student and retired)	12 (2.3)
Undisclosed	4 (12.9)
Annual household income	
< $50 000	7 (22.6)
$50 000–$99 999	12 (38.7)
> $100 000	6 (19.4)
Undisclosed	4 (12.9)
Highest level of education	
High school	6 (19.4)
College or technical school	6 (19.4)
University, undergraduate (e.g., Bachelors)	11 (35.5)
University, graduate (e.g., Masters, PhD and MD)	7 (22.6)
Undisclosed	1 (3.2)

Child demographic profile	*n* (%)
Age (years), M (SD)	4.0 (0)
Gender	
Girl	11 (35.5)
Boy	20 (64.5)
Ethnicity	
White	18 (60.0)
Asian	4 (12.9)
Indigenous	3 (9.7)
Mixed race/other	5 (16.1)
Undisclosed	1 (3.2)
Disability group	
Developmental	17 (54.8)
Physical	4 (12.9)
Sensory	3 (9.7)
Mental health	1 (3.2)
Multiple	6 (19.4)

**TABLE 2:** Descriptive analyses of children's daily PA, screen time and sleep behaviours.

	M	SD	Min	Max
Average daily minutes of leisure time PA^a^	108.3	89.9	0.0	270.4
Light intensity^a^	74.2	69.7	0.0	137.5
Moderate intensity^a^	21.9	23.6	0.0	85.7
Vigorous intensity^a^	13.6	16.0	0.0	51.4
Moderate‐to‐vigorous intensity^a^	40.1	47.0	0.0	188.6
Average daily minutes of screen time^b^	224.8	169.2	0.0	694.3
Video games^c^	60.7	77.2	0.0	291.4
TV^c^	82.5	71.4	0.0	291.4
Computer^c^	39.8	51.9	0.0	154.3
Phone^b^	47.4	52.8	0.0	197.1
Average nightly hours of sleep^b^	10.6	1.0	8.3	12.1

Abbreviations: PA = physical activity, M = mean, SD = standard deviation.

Sample sizes:

^a^ *n* = 26.

^b^ *n* = 29.

^c^ *n* = 28.

## 4 Discussion

This study provides insights into the 24‐h movement behaviours of preschool‐aged children with disabilities in a sample of preschool‐aged children living in Canada. Similar to results presented for preschool‐aged children without disabilities (Cliff et al. 2017; Nyström et al. 2020; Santos et al. 2017; Tanaka et al. 2023) and older children and youth with disabilities (Arbour‐Nicitopoulos et al. 2022; Law et al. 2013; Ng et al. 2023), guideline adherence rates are lowest for sedentary behaviour and highest for sleep. With regard to PA, children in this study were engaging in, on average, 72 min less of daily PA than what is recommended for their chronological age, and fewer children met the age‐specific PA guideline compared with studies of preschool‐aged children without disabilities (Cliff et al. 2017; Tanaka et al. 2023). In Canada, preschool‐aged children without disabilities have been reported to engage in approximately 1.8 h of MVPA per day (Kuzik et al. 2020) and adherence rates for the PA guideline have been shown to be as high as 62% (Chaput et al. 2017). In contrast, children in this study engaged in an average of 40 min of MVPA per day and only 23% of children met the PA guideline. Previous research has shown that the benefits of PA may be amplified for children and adults with disabilities (Anderson and Heyne 2010). Although the sample of young children with disabilities in this study was reported to be engaging in less PA than what is recommended for preschool‐aged children, they may still be experiencing meaningful health benefits from the PA they participate in (Anderson and Heyne 2010). Specifically, children in this study were reported to be engaging in over an hour (i.e., 108 min) of daily PA at any intensity, with most of those PA minutes being spent at a light intensity. Engaging in light‐intensity PA has been shown to be positively related to health outcomes in school‐aged children and adults and is therefore important to maintain (Poitras et al. 2016; Ross et al. 2024). Thus, in addition to mobilizing efforts to increase MVPA among young children with disabilities, equal efforts should be directed towards increasing and maintaining participation in light‐intensity PA.

The current sample of preschool‐aged children with disabilities were reported to engage in over three times the recommended levels of recreational screen time, and only 8% met the guideline of less than 60 min of screen time per day. This finding partially aligns with the results from studies of preschool‐aged children without disabilities, which report adherence rates for meeting the sedentary behaviour guideline of 11% (Santos et al. 2017) to 38% (Nyström et al. 2020). The lower adherence rate for the sedentary behaviour guideline reported in the current study aligns with previous research showing that older children and youth with disabilities engage in more recreational screen time than children and youth without disabilities (Law et al. 2013). Previous studies have shown that children with disabilities in the early years spend most of their time participating in activities in the home environment, with a preference for screen‐based activities (Di Marino et al. 2018). This may, in part, be due to a lack of inclusive and accessible activities available for families with young children with disabilities to participate in (Bassett‐Gunter et al. 2017; Bedell et al. 2013; Shields et al. 2012). Increasing opportunities for preschool‐aged children with disabilities and their families to participate within the community, away from screens, may help to reduce recreational screen time while also increasing PA participation among families.

This study is the first to examine all three movement behaviours among preschool‐aged children with disabilities and thus begins to address the current gap in movement behaviour research in this population (Arbour‐Nicitopoulos et al. 2022). However, the small sample of primarily boys from two provinces in Canada (British Columbia and Ontario) limits the generalizability of the results to other young children with disabilities living in Canada. Additionally, all participants were 4 years old, warranting future exploration of movement behaviours among younger preschool‐aged children, toddlers and infants. The use of previously validated measures of movement behaviours among children without disabilities is a strength of this study; however, the use of these tools for children with disabilities may have influenced the reliability and validity of the findings, in addition to the reliance on caregiver‐reported data, which may introduce reporting bias. Future studies should incorporate measures such as accelerometers or observational methods to more accurately capture young children's movement behaviours.

In conclusion, results from this study suggest low adherence rates among preschool‐aged children with disabilities for meeting the sedentary behaviour and PA guidelines. These findings underscore the critical need for targeted interventions to improve PA and reduce sedentary behaviour in young children with disabilities, with future research necessary to confirm these results in a larger more representative sample and to explore participation patterns across age groups, sexes and disability types in the early years.

## Author Contributions

**Maeghan E. James:** conceptualization, writing – original draft, methodology, writing – review and editing, formal analysis, project administration. **Kathleen A. Martin Ginis:** conceptualization, funding acquisition, methodology, writing – review and editing. **Rebecca L. Bassett‐Gunter:** conceptualization, funding acquisition, methodology, writing – review and editing. **Krista L. Best:** conceptualization, funding acquisition, methodology, writing – review and editing. **Kelly P. Arbour‐Nicitopoulos:** supervision, formal analysis, methodology, conceptualization, funding acquisition, writing – original draft, writing – review and editing, project administration.

## Conflicts of Interest

The authors declare no conflicts of interest.

## Data Availability

The data that support the findings of this study are available from the corresponding author upon reasonable request.

## Survey Items

The following table outlines the questions and corresponding response options used to measure the 24‐h movement behaviours in this study.

**Table:**

Movement behaviour	Question	Response option	Method for calculating total time and determining guideline adherence
Leisure time physical activity^a^	In the last 7 days, on how many days did your child [walk or wheel, not as a means of transportation/do moderate PA/do vigorous PA] in their leisure time?	0 days 1 day 2 days 3 days 4 days 5 days 6 days 7 days Prefer not to answer	An average duration of daily minutes of each PA intensity was calculated by multiplying frequency by duration and dividing by 7 days. Total daily PA was calculated by summing the average daily minutes spent in light, moderate and vigorous leisure time PA. To determine PA guideline adherence, total average daily minutes of leisure time total PA and minutes of moderate‐to‐vigorous PA (MVPA) were used. Children met the PA guideline for preschool‐aged children if their caregiver reported that the child engaged in an average of 180 min of total PA per day, of which 60 min were spent engaging in leisure time MVPA.
	How much time did your child usually spend on one of those days [walking or wheeling/doing moderate PA/doing vigorous PA] in their leisure time? Please estimate the number of hours and/or minutes on one of those days. For example, if your child spent 1 and a half hours walking or wheeling during their leisure time enter ‘1’ under hours and ‘30’ under minutes.	Hours: _____ Minutes: ____	
Recreational screen time^b^	On [weekdays/weekends], about how many hours a day does your child usually play games on a computer or games console (e.g., on a tablet or smartphone, PlayStation, Xbox, GameCube)/in their free time?	None at all Half an hour a day 1 h a day 2 h a day 3 h a day 4 h a day 5 h a day 6 h a day 7 or more hours a day Prefer not to answer	A weighted daily average for total screen time and individual types of screen time was calculated by summing the average weekday hours (weekday hours/5 days) and average weekend hours (weekend hours/2 days) and dividing by seven total days. Children met the screen time guideline if their caregiver reported that they engaged in less than 1 h per day of recreational screen time.
	On [weekdays/weekends], about how many hours a day does your child usually watch television (including DVDs in their free time?	None at all Half an hour a day 1 h a day 2 h a day 3 h a day 4 h a day 5 h a day 6 h a day 7 or more hours a day Prefer not to answer	
	On [weekdays/weekends], about how many hours a day does your child usually use a computer for entertainment purposes (e.g., watching YouTube videos, social media, chatting online and browsing the Internet) in their free time?	None at all Half an hour a day 1 h a day 2 h a day 3 h a day 4 h a day 5 h a day 6 h a day 7 or more hours a day Prefer not to answer	
	On [weekdays/weekends], about how many hours a day does your child usually use their smartphone or tablet (if they have one) in their free time?	None at all Half an hour a day 1 h a day 2 h a day 3 h a day 4 h a day 5 h a day 6 h a day 7 or more hours a day Prefer not to answer	
Sleep^c^	During the past week, what time does your child typically wake up on [school days/weekend days]? Please indicate the time as hh:mm.	[open text]	Total sleep duration was calculated using the time the child went to bed and the time they woke the next morning. A weighted daily average for sleep was calculated. Children met the sleep guideline if they slept, on average, between 10 and 13 h per night.
	During the past week, what time does your child typically turn out the lights and go to sleep on [school days/weekend days]? Please indicate the time as hh:mm.	[open text]	

^a^ Items derived from the International Physical Activity Questionnaire for Adolescents (IPAQ‐A).

^b^ Items derived from the Health Behaviour in School‐aged Children survey.

^c^ Items derived from the International Study on Childhood Obesity, Lifestyle, and the Environment (ISCOLE) Diet and Lifestyle Questionnaire.
